# Target identification for the diagnosis and intervention of vulnerable atherosclerotic plaques beyond ^18^F-fluorodeoxyglucose positron emission tomography imaging: promising tracers on the horizon

**DOI:** 10.1007/s00259-018-4176-z

**Published:** 2018-10-09

**Authors:** Jan Bucerius, Ingrid Dijkgraaf, Felix M. Mottaghy, Leon J. Schurgers

**Affiliations:** 1 Department of Radiology and Nuclear Medicine, Maastricht University Medical Center (MUMC+), 6229 HX Maastricht, The Netherlands; 2 Cardiovascular Research Institute Maastricht (CARIM), Maastricht University Medical Center (MUMC+), 6200 MD Maastricht, The Netherlands; 3 Department of Nuclear Medicine, University Hospital RWTH Aachen, Aachen, Germany; 4 Department of Biochemistry, Maastricht University, Maastricht, The Netherlands

**Keywords:** Atherosclerotic plaque, PET tracers, Cardiovascular disease, Vascular smooth muscle cells, Vascular calcification

## Abstract

Cardiovascular disease is the major cause of morbidity and mortality in developed countries and atherosclerosis is the major cause of cardiovascular disease. Atherosclerotic lesions obstruct blood flow in the arterial vessel wall and can rupture leading to the formation of occlusive thrombi. Conventional diagnostic tools are still of limited value for identifying the vulnerable arterial plaque and for predicting its risk of rupture and of releasing thromboembolic material. Knowledge of the molecular and biological processes implicated in the process of atherosclerosis will advance the development of imaging probes to differentiate the vulnerable plaque. The development of imaging probes with high sensitivity and specificity in identifying high-risk atherosclerotic vessel wall changes and plaques is crucial for improving knowledge-based decisions and tailored individual interventions. Arterial PET imaging with ^18^F-FDG has shown promising results in identifying inflammatory vessel wall changes in numerous studies and clinical trials. However, due to its limited specificity in general and its intense physiological uptake in the left ventricular myocardium that impair imaging of the coronary arteries, different PET tracers for the molecular imaging of atherosclerosis have been evaluated. This review describes biological, chemical and medical expertise supporting a translational approach that will enable the development of new or the evaluation of existing PET tracers for the identification of vulnerable atherosclerotic plaques for better risk prediction and benefit to patients.

## Introduction

Vascular disease is still the leading cause of morbidity and mortality in the Western world, and the primary cause of myocardial infarction, stroke, and ischaemia. Atherosclerosis is a chronic disease of the large arteries that remains asymptomatic for decades. Arterial wall changes in the context of atherosclerosis start in childhood and vascular lesions enlarge with age and typically become symptomatic at 50–60 years of age. The pathobiology of atherosclerosis is complex and lesions can be generally divided into stable and unstable (also termed vulnerable) plaques. While stable plaques can occlude the lumen of the arterial vessel over time, they can develop plaque erosion. Moreover, plaque rupture is the dominant initiating event, responsible for 60–70% of acute coronary syndromes (ACS). It is therefore important to develop tools that discriminate between plaques that become vulnerable and plaques that remain stable but display erosion. State-of-the-art imaging using novel probes identifying molecular and cellular processes associated with plaque rupture have the potential for better patient stratification and personalized strategies.

## Plaque biology: cellular and molecular mechanisms

The process of atherogenesis starts with activation of vascular endothelial cells, either by vascular shear stress [1] or local processes [2]. Also platelets can deposit so-called footprints that direct leucocytes to the vasculature [3]. At the same time as leucocyte infiltration, a heterogeneous population of vascular smooth muscle cells (VSMCs) is established in the subintimal space [4]. It has been shown that VSMCs are important in health and disease [5]. The pool of intimal VSMCs in the atherosclerotic lesion might arise from the infiltration of haematopoietic stem cells that differentiate locally into VSMCs [6]. However, more recently, VSMC lineage tracing in apoE-null mice has shown that a majority of atherosclerotic lesion cells are derived from VSMCs from the vasculature [7]. The accumulation of low-density lipoproteins (LDL), that become oxidized, promotes macrophages to take up these remnants via their scavenger receptor. These foam cells are the starting point of the so-called fatty streak. In response to these proinflammatory macrophages, more VSMCs migrate into the vascular intima producing a fibrous cap around the xanthomal lesion. In addition, the proinflammatory macrophages attract more macrophages which further fuel the inflammatory vascular process. Due to hypoxia in the core of the atherosclerotic plaque, a necrotic core develops and intraplaque neovascularization is upregulated in response. Finally, cellular debris calcifies and the amount of calcification is a measure of the atherosclerotic burden [8].

Many features have been linked to plaque vulnerability including perivascular inflammation, large necrotic core, thin fibrous cap, microcalcifications, intraplaque haemorrhage, neoangiogenesis and hypoxia [9]. A thin fibrous cap is the result of degradation of collagen by metalloproteinase derived from activated macrophages. The rupture of an atherosclerotic plaque usually takes place at the shoulders of the plaque where the cap is thinnest. A large necrotic core and increased angiogenesis are a consequence of inflammation and hypoxia. Endothelial cells of intraplaque (micro)vessels express adhesion molecules favouring leucocyte recruitment. These angiogenic vessels are immature and fragile and due to extravasation of leucocytes further contribute to atherosclerosis progression. Inflammation has been linked to both apoptosis and calcification [10, 11], and oxidative stress has been shown to be responsible for VSMC calcification [12, 13].

Vascular calcification was long considered a passive process not amenable to intervention. A paradigm shift came with the discovery that so-called Gla proteins are involved in the regulation of mineral deposition in the vasculature [14]. Vascular calcification is now considered an active process with cellular and humoral contributions which plays an important role in cardiovascular morbidity and mortality [15]. The discovery that vitamin K-dependent proteins are inhibitors of vascular calcification has advanced our mechanistic understanding of the calcification process and opened novel avenues for diagnosis and treatment [16].

Vascular calcification comes in different flavours: calcification can occur in the vascular intima as well as in the media and it may have different shapes and sizes. Recent data suggest that microcalcification can have a destabilizing effect on atherosclerotic plaques [2, 17], in contrast to the hypothesis that large calcifications are plaque-stabilizing. Microcalcification arises from dedifferentiated VSMCs that start secreting extracellular vesicles, that in the extracellular space form the nidus for calcification [18]. These microcalcifications, which are undetectable by conventional imaging, increase the local stress inside the thin fibrous cap twofold and thus increase the likelihood of rupture [19]. These microcalcifications will eventually result in macrocalcification as a product of osteogenic action by osteocyte-like and chondrocyte-like cells inside the plaque. Novel imaging tools allow the course of microcalcification to be followed [20]. In a rodent model of atherosclerosis, macrophage infiltration in early atherosclerotic plaques has been shown to colocalize with calcification. Indeed, microcalcifications that are phagocytosed by macrophages polarize towards the proinflammatory M1 phenotype [21].

Effective screening to identify the vulnerable plaque is still lacking. This is largely due to the absence of insight into the pathophysiology of vulnerable plaque and thus the lack of cost-effective interventions. Since the genesis of microcalcification is considered one of the earliest precursor processes in the formation of vulnerable plaque [2, 22], adequate early intervention and prevention are possible. Therefore, it is important to understand the factors that drive microcalcification as well as the underlying mechanisms. However, the vast majority of so-called ‘vulnerable’ plaques do not exhibit clinical ‘instability’ and indeed seldom induce ACS [23]. Due to effective treatment, i.e. with statins, lesions became more stable. These lesions underlying superficial erosion do not have thin fibrous caps, have fewer inflammatory cells and lack a large lipid core. Rather they are more collagen-rich lesions and have more calcification, but they are still prone to form occlusive thrombi [23]. These considerations, taken together, indicate the high promise of vascular calcification as a new target for diagnosis and intervention in cardiovascular diseases, with an urgent need to translate experimental findings into adequate diagnostic, preventive and therapeutic solutions.

## ^18^F-FDG PET imaging of atherosclerosis

The strength of nuclear medicine is its ability to provide quantitative information at a functional level, such as the density of a specific receptor or the metabolic activity of a plaque. Nuclear imaging is based on radiolabelled biomarkers with a signal sensitivity in the picomolar range, which compares favourably with both MRI and especially CT, both of which have sensitivities up to a trillion times lower [21, 22]. PET images are derived from the detection of positron-emitting radionuclides labelling biochemical and metabolic substrates. The radionuclide is administered intravenously and circulates within the body. This allows time for the tracer to accumulate at the site of interest. To obtain a favourable target-to-background ratio, the radionuclide should be cleared from the blood quickly. The high sensitivity of PET coupled with the favourable resolution of CT and MRI, as already achieved in PET/CT and PET/MRI hybrid imaging systems provides valuable information about the localization of the acquired signal [24–26].

To obtain good quality images a high target-to-background ratio is mandatory for vascular PET imaging, for which the radiotracer must show high uptake in the target and preferably also rapid clearance from the bloodstream. Due to the small size of plaques, a high background activity mainly in the blood would severely impair the quality of PET images. Furthermore, coronary artery imaging presents special problems. Cardiac and respiratory movement, myocardial tracer uptake, especially of ^18^F-FDG, and the small size (3 to 4 mm) of the coronary arteries may impair and even preclude imaging of the coronary arteries by PET. Whereas motion artefacts can be reduced by using modalities such as cardiac gating, especially with the relatively slow PET data acquisition, suppression of the unwanted myocardial ^18^F-FDG uptake might be achieved using a different available dietary protocols [21, 22].

The radioactive tracer most commonly used clinically for PET imaging is ^18^F-FDG. ^18^F-FDG is mainly used for oncological studies and is well-established for the diagnosis and staging of malignant tumours and the monitoring of therapy response. The tracer is transported into cells by glucose transporters and is phosphorylated by hexokinase to ^18^F-FDG-6-phosphate that is not further metabolized [27]. The degree of cellular ^18^F-FDG uptake is related to the metabolic rate and the number of glucose transporters [28]. Therefore, increased ^18^F-FDG uptake in malignant tumour cells is partly due to an increased number of glucose transporters found in these cells. An increased number of glucose transporters is also found in activated inflammatory cells, such as macrophages. Additionally, the affinity of glucose transporters for deoxyglucose is apparently increased in these cells under inflammatory conditions by various cytokines and growth factors [27, 29]. However, potential competition between ^18^F-FDG and nonradioactive stable glucose for uptake in metabolically active cells (for example, inflammatory) might affect the imaging results in patients with high prescan glucose levels. Furthermore, an excess of unlabelled glucose and the action of insulin may increasingly affect the accumulation of ^18^F-FDG due to saturation of the glucose transporters [30, 31]. In addition, increases in plasma insulin levels result in translocation of the GLUT-4 transporters from an intracellular pool to the plasma membrane [32, 33]. This may enhance the uptake of ^18^F-FDG in myocardial and skeletal muscle, which in turn could significantly impair the quality and interpretability of ^18^F-FDG PET scans. This issue needs to be kept in mind particularly in patients with diabetes who are frequently affected by atherosclerosis, as diabetes is one of the main cardiovascular risk factors [34–39].

## ^18^F-FDG PET in preclinical studies

### Methodological considerations of preclinical PET imaging of atherosclerosis

The availability of dedicated small-animal PET as well as hybrid PET/CT and now PET/MRI systems offers and facilitates the widespread use of preclinical PET imaging of atherosclerosis. This is crucial not only to understand the pathophysiology of the development and progression of atherosclerosis in vivo, but also to evaluate newly developed PET tracers for functional imaging of atherosclerosis. This also applies to PET tracers originally introduced into clinical routine for other indications, for example ^68^Ga-DOTATATE for the imaging of neuroendocrine tumours and ^18^F-fluorocholine (^18^F-FCH) for the (former) imaging of prostate cancer disease. To investigate their ability to enhance the visibility of atherosclerosis on PET they can be tested in vivo in a preclinical setting and correlated with histology for imaging distinct parts of the disease process.

The most common tool used in preclinical studies of atherosclerosis is still genetically modified mouse models [40]. Using these mouse models has provided a deep insight into the pathophysiological mechanisms in atherosclerosis despite obvious and well-known differences between human and murine atherosclerosis – including vessel architecture, plaque composition and stability [40–42]. The currently available genetically modified mouse models are suitable for initial evaluation of new PET tracers for atherosclerosis imaging. However, several steps of the human atherosclerotic disease process still cannot be covered by the available preclinical mouse models, impairing comparison with and translation to the human situation [40]. Therefore, attempts are being made to develop more appropriate mouse models to study plaque rupture [43]. Suggested models are brachiocephalic plaque disruption in aged apoE-null mice fed a high fat diet [44], plaque rupture in the brachiocephalic artery in mice fed a high fat diet and receiving angiotensin II [45] or warfarin [46], transfection of mice with haematopoietic stem cells with overexpression of matrix metalloproteinase 9 (MMP-9) [47] or urokinase plasminogen-activator (uPA) in macrophages [48], or collar placement on the carotid artery in apoE-null mice with stimulation with lipopolysaccharide and phenylephrine [49]. The suitability of these mouse models for mimicking atherosclerosis in humans more closely has to be proven in the near future.

Another approach to overcoming the shortcomings of atherosclerotic mouse models is the use of larger species for preclinical imaging such as rabbit or porcine models. Because of their greater size, rabbit arteries are large enough for more detailed analysis of arterial plaque components by PET, MRI and CT. This also allows serial imaging analysis in the context of interventional studies testing for strategies in plaque stabilization and/or regression, for example by applying lipid-lowering drugs. The size of rabbit arteries also allows the placement of, for example, endovascular stents, a model which can be used for the assessment of re-endothelialization of vascular therapeutic devices measured using PET [50, 51].

Recently, a rabbit model of vulnerable atherosclerotic plaques has been introduced, and seems to overcome the absence of an appropriate mouse model for this purpose. This model consists of placing a vascular (arterial) injury in rabbits receiving a hyperlipidaemic diet [52–54]. Of all preclinical models representative of the clinical situation, porcine models may be the best option for the investigation of atherosclerosis. Pigs not only exhibit vessel wall architecture similar to that in humans, but also respond in the same way as humans to exposure to oxidized LDL. Furthermore, they develop atheroma lesions in response to insulin resistance and also develop vulnerable atherosclerotic plaques [43, 55, 56]. Besides the pathophysiological similarities between porcine and human plaques, another advantage of using porcine atherosclerosis models is related to the size of the porcine vessels, which are sufficiently large to enable the evaluation of not only small vessels such as the coronary arteries but also neointimal hyperplasia and neovascularization. Furthermore, PET imaging of smaller arterial structures is also facilitated by the larger vessel size, which is crucial because of the rather low spatial resolution of PET compared with that of CT and MRI [57].

From a technical point of view, the combination of noninvasive imaging methodologies such as PET and CT or MRI facilitates efforts to develop new preclinical models for atherosclerosis imaging. Such models would allow not only evaluation of the functional aspects of a ‘vulnerable’ atherosclerotic plaque with PET, but also determination of the precise location as well as morphological parameters of atherosclerotic lesions with CT or MRI.

Unquestionably, animal models are indispensable to the elucidation of the molecular mechanisms that underlie human atherosclerosis and plaque vulnerability. Each animal model has its own advantages and disadvantages concerning cost and the possibility of modulating cell and cellular factors that influence the atherogenic process. In order to answer a research question properly, the correct choice of animal model is decisive.

### Preclinical imaging studies with ^18^F-FDG PET

Preclinical studies indicating that ^18^F-FDG PET might have a role in imaging atherosclerosis have been performed in cholesterol-fed rabbits. A positron-sensitive fibre-optic probe placed in contact with the arterial intima was able to detect high ^18^F-FDG uptake in atherosclerotic segments of the iliac artery [58]. That ^18^F-FDG is able to identify vulnerable plaque was demonstrated in a rabbit model of atherosclerosis in which plaque rupture was promoted by venom injection. Aziz et al. found that only the aortic plaques with the highest preinjection ^18^F-FDG uptake progressed to rupture and thrombosis [59]. The suitability of ^18^F-FDG PET for the investigation of therapeutic interventions was shown in a rabbit model of atherosclerosis in which there was a significant reduction in ^18^F-FDG uptake after 3 months of therapy with a lipid-lowering antioxidant agent [60].

Imaging atherosclerosis with ^18^F-FDG PET also has potential drawbacks. ^18^F-FDG was administered intravenously to atherosclerotic LDLR/ApoB48 mice and control mice and the ex vivo distribution in frozen aortic sections was measured by digital autoradiography [61]. Additionally, in vitro uptake of ^18^F-FDG in human atherosclerotic arteries was also examined. Unexpectedly, significant ^18^F-FDG uptake was found in the vicinity of calcified structures atherosclerotic plaques in mice. This uptake seemed to be more prominent than in noncalcified atherosclerotic plaques. The in vitro studies of atherosclerotic human arteries showed similar results with marked uptake of ^18^F-FDG in the calcifications but not in other structures of the artery wall.

To overcome the limitations of ^18^F-FDG PET imaging of atherosclerosis, PET tracers originally introduced for imaging other indications (such as oncological or neurological disorders) have been used in numerous preclinical studies to evaluate potential candidates for screening of vulnerable atherosclerotic plaque. In these studies, different PET tracers were used to those used in preclinical models; for example, ^18^F-FCH and ^18^F-labelled analogues of CGS 27023A for imaging MMPs, and ^124^I-labelled platelet glycoprotein VI (^124^I-GPVI) for imaging thrombosis at sites of atherosclerotic lesions [62–65].

## ^18^F-FDG PET imaging in clinical studies

Arterial ^18^F-FDG uptake was first noted in the aorta of patients undergoing ^18^F-FDG PET for cancer imaging [66]. It was also shown that the amount of ^18^F-FDG uptake increased with age and was higher in patients with cardiovascular risk factors [29, 67, 68]. Supported by cell culture work, ^18^F-FDG uptake in the arterial wall can probably be attributed to uptake in plaque macrophages. These studies demonstrated that increased oxidative metabolism and glucose use in response to cellular activating agents is accompanied by a dramatic increase in ^18^F-FDG uptake in both leucocytes and macrophages [36, 69].

Following the dedicated preclinical studies of the use of ^18^F-FDG PET for imaging atherosclerosis, Rudd et al. performed one of the first clinical studies of atherosclerosis imaging with ^18^F-FDG PET in patients with transient ischaemic attack [70]. The patients were scanned shortly after symptom onset and a significantly higher ^18^F-FDG uptake (almost 30%) was seen in symptomatic carotid plaques than in the asymptomatic artery [70]. This indicates the feasibility of ^18^F-FDG PET for the imaging of inflammation in the aorta, and in peripheral and vertebral arteries [71–73]. The relationship between uptake of ^18^F-FDG and inflammation has been demonstrated using histology linking ^18^F-FDG uptake with the number of macrophages in arterial specimens [74, 75]. In addition to findings indicating ^18^F-FDG uptake as a marker of arterial inflammation, preliminary studies have indicated that ^18^F-FDG has the potential to predict plaque rupture as well as clinical events. Regarding the predictive value of ^18^F-FDG PET, in a study including more than 2,000 patients with cancer disease, Paulmier et al. found that, compared with patients with a low arterial ^18^F-FDG uptake, patients with the highest ^18^F-FDG uptake were more likely to have either a previous vascular event or to experience an event during the 6 months after PET imaging [76]. Furthermore, the short-term interscan reproducibility of ^18^F-FDG PET in the imaging of atherosclerosis has been shown to be excellent for the carotid artery, aorta and peripheral arteries [69, 71, 73, 77]; this is an important finding mainly for the use of ^18^F-FDG PET as an endpoint parameter in intervention studies.

Despite the widespread use of ^18^F-FDG PET/CT in cardiovascular disease, nonspecific uptake of the tracer is undoubtedly the most important drawback of the use of ^18^F-FDG for imaging atherosclerosis [54]. ^18^F-FDG is nonspecifically taken up by almost all human tissues and organs to a certain degree. ^18^F-FDG uptake analysis in arterial and/or venous vessels is therefore hampered by significant spill-over of tracer signal from closely neighbouring structures; for example, ^18^F-FDG uptake in the thyroid gland during carotid PET imaging or myocardial ^18^F-FDG uptake during PET imaging of the thoracic aorta or coronary arteries. Furthermore, pathologically increased ^18^F-FDG uptake in structures close to the target vessel further impair ^18^F-FDG uptake analysis in atherosclerotic plaques; for example, inflamed cervical lymph nodes close to the carotid arteries. More importantly, significant ^18^F-FDG uptake has been found in atherosclerosis-prone mice in the vicinity of calcified structures in plaques, and this has been confirmed in in vitro studies of atherosclerotic human arteries that showed marked binding of ^18^F-FDG to calcifications but not to other structures of the artery wall [61]. On the basis of these findings taken together, studies to evaluate new or already existing PET tracers currently used for indications other than atherosclerosis are warranted. These PET tracers might be more specific and possibly more sensitive for detecting high-risk vulnerable plaques.

## Beyond ^18^F-FDG: PET radiotracers for target identification in atherosclerosis

### ^18^F-Fluorocholine

^18^F-FCH was introduced several years ago for imaging the brain and for diagnosis of prostate cancer disease [78, 79]. Choline is taken up into cells by specific transport mechanisms and phosphorylated by choline kinase. Increased choline uptake has been shown both in tumour cells and in activated macrophages [80, 81]. Previously published studies demonstrated enhanced ^18^F-FCH uptake after soft tissue infection or acute cerebral radiation injury that correlated well with macrophage accumulation as part of an inflammatory reaction [82, 83]. In a murine model of atherosclerosis, ex vivo imaging with ^18^F-FCH provided better identification of plaques than imaging with ^18^F-FDG, indicating that this tracer may be a promising candidate for imaging plaques in patients [62].

The first feasibility study of the use of ^18^F-methylcholine (^18^F-FMCH) PET for imaging arterial plaques was performed in five patients undergoing imaging for prostate cancer [84]. Morphological classification of vessel wall alterations in the abdominal aorta and the common iliac arteries included structural wall alterations without additional calcifications, structural wall alterations associated with calcifications, and solely calcified lesions. The use of ^18^F-FMCH was shown to be feasible for in vivo imaging of structural vessel wall alterations in humans, which provided the basis for several subsequent studies on vascular ^18^F-FCH PET imaging [84]. Confirming these results, Kato et al. reported the results of a retrospective study of the use of ^11^C-choline PET imaging of vascular plaques in 93 patients with prostate cancer [85]. ^11^C-Choline uptake was found in 95% of the patients and calcification in 94% throughout all vessel segments. However, colocalization of choline uptake with calcifications was found in only 6% of the patients. Furthermore, less than 1% of calcification sites showed increased radiotracer uptake. Thus, in concordance with the previous data, ^11^C-choline uptake and calcification were found to be only rarely colocalized [84, 85]. Because of the high uptake of ^11^C-choline and the high proportion of calcifications without colocalization, ^11^C-choline potentially provides information about atherosclerotic plaques independent of calcification measurement [85].

In a retrospective study including 60 patients with prostate cancer examined with whole-body PET/CT using ^18^F-fluoroethylcholine (^18^F-FEC), uptake in the wall of the ascending and descending aorta, aortic arch, abdominal aorta, and both iliac arteries was measured, and the sum of calcified plaques (CP_sum_) in these vessels was also calculated. These data were correlated with the presence of cardiovascular risk factors and occurrence of prior cardiovascular events [86]. CP_sum_ was significantly correlated with cardiovascular risk factors. However, no significant association was found between ^18^F-FEC uptake in large vessels and atherosclerotic plaque burden, in contrast to the studies by Bucerius et al. and Kato et al. [84, 85]. It is noteworthy that the lack of an association between choline uptake and calcified arterial plaque burden reported by Förster et al. was also seen in the two previous studies of vascular choline PET imaging [84–86]. Recently, a prospective study including ten consecutive stroke patients with ipsilateral carotid artery stenosis of >70% was reported. Patients were scheduled for carotid endarterectomy and underwent PET for the assessment of the maximum ^18^F-FCH uptake in the ipsilateral symptomatic carotid plaques and contralateral asymptomatic carotid arteries [87]. The PET imaging results were correlated with histological evaluation of the macrophage content in all carotid endarterectomy specimens as the CD68+ staining percentage per whole plaque area and as the maximum CD68+ staining percentage in the most inflamed section/plaque. A strong correlation between ^18^F-FCH uptake in the carotid atherosclerotic plaque and degree of macrophage infiltration indicates that ^18^F-FCH PET is a promising tool for the evaluation of vulnerable plaques [87].

### ^68^Ga-DOTATATE

**Fig. 1:**
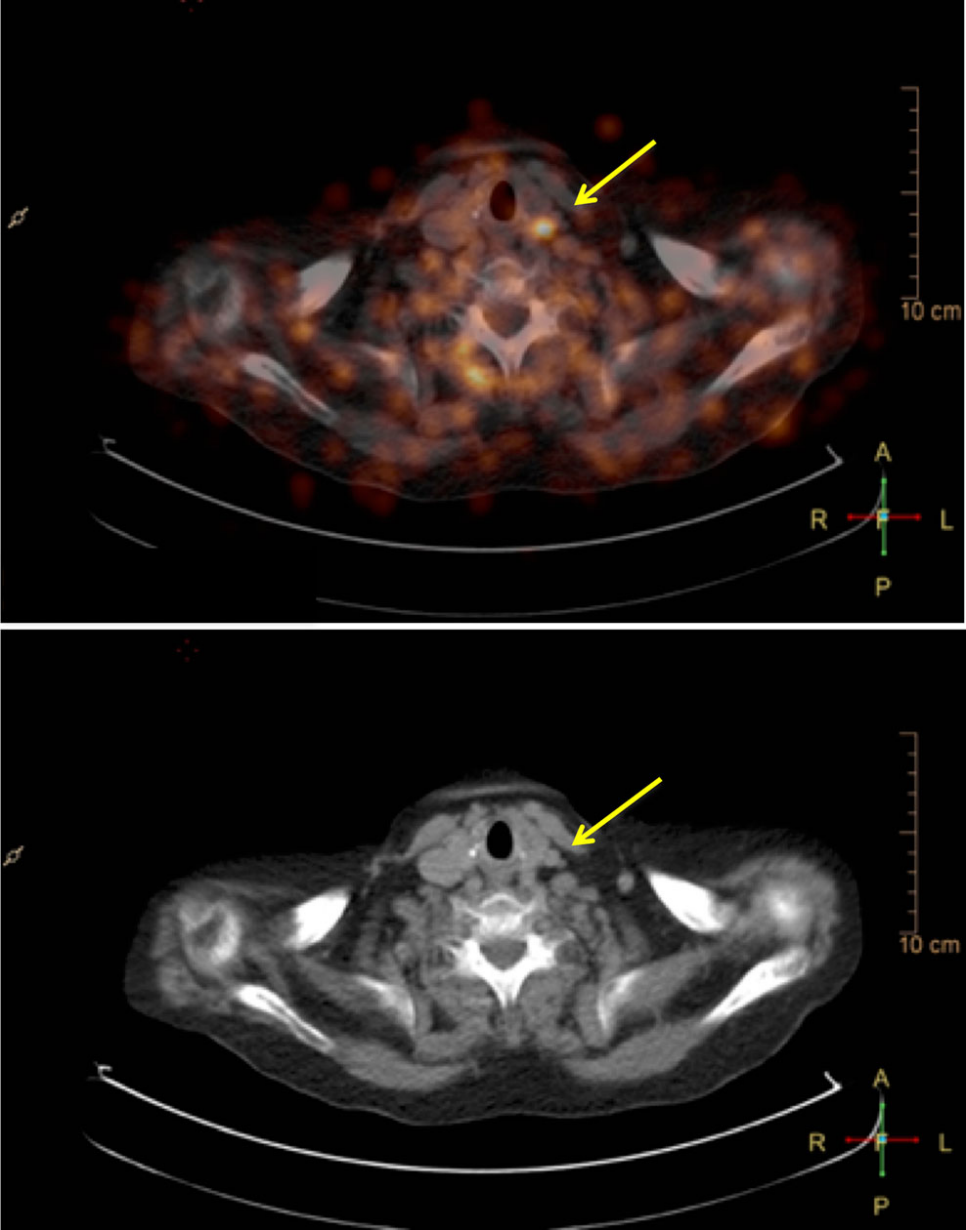
Fused PET/CT and CT images show ^68^Ga-DOTATATE uptake in the left common carotid artery close to the bifurcation (*arrows*), which is visually and semiquantitatively higher than in the right carotid artery, indicating increased inflammatory changes in the left carotid artery

**Fig. 2:**
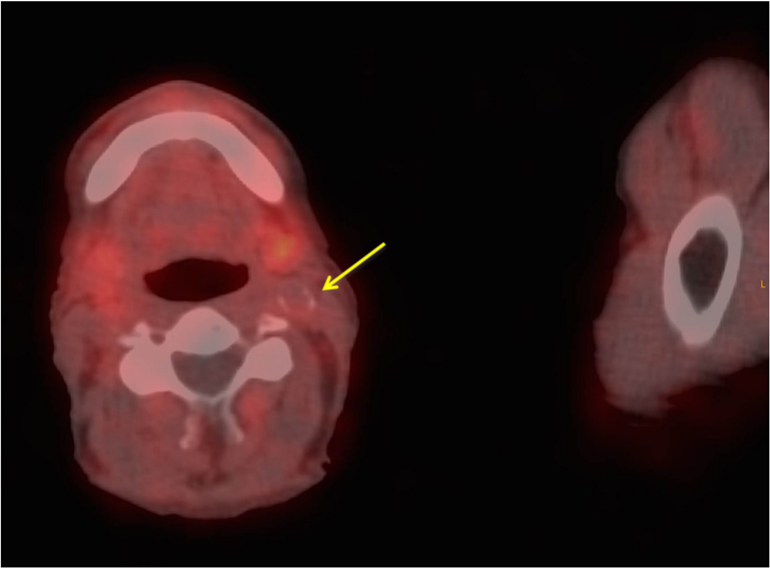
Fused PET/CT images show only slight ^68^Ga-DOTATATE uptake at the bifurcation of the left carotid artery with, on the CT images, as part of the fused PET/CT images, visible calcification (*arrow*). Combined molecular (^68^Ga-DOTATATE PET) and morphological imaging (CT) indicates more stable, chronic arterial vessel wall changes

^68^Ga-DOTATATE PET imaging has gained widespread acceptance in clinical routine for imaging somatostatin receptor-positive neuroendocrine tumours. These somatostatin receptors of subtype 2 are also expressed by macrophages and can therefore be detected by ^68^Ga-DOTATATE PET imaging [88–90]. One advantage compared to ^18^F-FDG is that ^68^Ga-DOTATATE does not show physiological uptake in the myocardium. Therefore, besides imaging of the carotid arteries (Figs. 1 and 2) and aorta, imaging of the coronary arteries with ^68^Ga-DOTATATE seems feasible, independent of the still unresolved methodological issues such as inappropriate spatial and temporal resolution related to coronary PET imaging. Rominger et al, determined the uptake of ^68^Ga-DOTATATE in the left anterior descending artery (LAD) in a series of 70 oncological patients undergoing ^68^Ga-DOTATATE whole-body PET/CT [90]. ^68^Ga-DOTATATE uptake was detectable in the LAD of all patients and correlated significantly with the presence of calcified plaques and prior vascular events. Calcified plaque burden was also correlated with prior vascular events and with patient age and the presence of hypertension. Therefore, the use of ^68^Ga-DOTATATE seems feasible for the imaging of vulnerable atherosclerotic plaque in coronary arteries.

Li et al. compared the uptake of ^68^Ga-DOTATATE and ^18^F-FDG in eight arterial segments in 16 patients with neuroendocrine tumours or thyroid cancer who underwent PET imaging with both tracers for staging or restaging [91]. ^68^Ga-DOTATATE uptake in all large arteries was correlated significantly with the presence of calcified plaques, the presence of hypertension, age and uptake of ^18^F-FDG. In contrast, ^18^F-FDG uptake was significantly correlated only with the presence of hypertension. Of the 37 sites with the highest focal ^68^Ga-DOTATATE uptake, 16 (43.2%) also had focal ^18^F-FDG uptake. In contrast, of 39 sites with the highest ^18^F-FDG uptake, only 11 (28.2%) showed colocalization with ^68^Ga-DOTATATE uptake. Thus, ^68^Ga-DOTATATE uptake seems to be more strongly associated with known risk factors of cardiovascular disease than ^18^F-FDG uptake. Interestingly, focal uptake of ^68^Ga-DOTATATE was shown not to be colocalized with ^18^F-FDG uptake in a significant number of lesions [91]. The disparity between these two tracers seems to reflect the fact that ^68^Ga-DOTATATE is a specific macrophage marker in atherosclerosis in contrast to ^18^F-FDG, whereas ^18^F-FDG provides a nonspecific measurement of glucose metabolism of cells within the atherosclerotic plaque. Furthermore, ^68^Ga-DOTATATE might offer a more ‘focal’ approach in imaging atherosclerotic lesions than ^18^F-FDG [92].

^64^Cu-DOTATATE can also be used in combination with vascular hybrid PET/MRI imaging to evaluate atherosclerosis in the carotid arteries [93]. Significantly higher ^64^Cu-DOTATATE uptake was found in symptomatic plaques than in the contralateral carotid artery. In a subsequent analysis, a total of 62 plaque segments were assessed for gene expression of selected markers of plaque vulnerability. A univariate analysis comparing these results with ^64^Cu-DOTATATE uptake in vivo showed that CD163 and CD68 gene expression was correlated weakly although significantly with the mean standardized uptake values in the PET scans. In a multivariate analysis CD163 was correlated independently with the ^64^Cu-DOTATATE uptake, whereas CD68 was not, indicating that ^64^Cu-DOTATATE-PET might detect alternatively activated macrophages.

### ^18^F-Sodium fluoride

^18^F-NaF is a positron-emitting bone-seeking agent which reflects blood flow and remodelling of bone. Therefore, ^18^F-NaF has received attention in the field of imaging alterations in calcification in atherosclerotic plaques. In a feasibility study, Derlin et al. retrospectively evaluated imaging data from 75 patients undergoing whole-body ^18^F-NaF PET/CT [94]. ^18^F-NaF uptake was observed at 254 sites in 57 patients (76%) and calcification was observed at 1,930 sites in 63 patients (84%). Colocalization of radiotracer accumulation and calcification was observed in 223 areas of uptake (88%). Interestingly, only 12% of all arterial calcification sites showed increased radiotracer uptake, indicating that ^18^F-NaF might be much more sensitive in detecting so-called spotty calcifications than CT. The same group investigated ^18^F-NaF uptake in the common carotid arteries of neurologically asymptomatic patients with cardiovascular risk factors and carotid calcified plaque burden [95]. They included 269 oncological patients who underwent ^18^F-NaF PET/CT. ^18^F-NaF uptake in the common carotid arteries was observed at 141 sites in 94 patients (34.9%) and showed colocalization with calcification in all atherosclerotic lesions. ^18^F-NaF uptake was significantly associated with age, male sex, and the presence of hypertension and hypercholesterolaemia. The presence of calcified atherosclerotic plaques was correlated significantly with these risk factors and also with diabetes, history of smoking and prior cardiovascular events. Furthermore, a highly significant correlation was found between ^18^F-NaF uptake and the number of cardiovascular risk factors.

Intriguing results of a prospective clinical trial of vascular ^18^F-NaF PET imaging have been reported [96]. Both ^18^F-NaF and ^18^F-FDG PET/CT and invasive coronary angiography were performed in 40 patients with myocardial infarction and 40 patients with stable angina. ^18^F-NaF uptake was compared with histology in carotid endarterectomy specimens from patients with symptomatic carotid disease, and with intravascular ultrasonography in patients with stable angina. In 37 patients (93%) with myocardial infarction, the highest coronary ^18^F-NaF uptake was seen in the culprit plaque. Interestingly, in contrast to the findings for ^18^F-NaF (which is not physiologically taken up by the myocardium), coronary ^18^F-FDG uptake was commonly obscured by myocardial uptake and where discernible, there were no differences between culprit and nonculprit plaques. At the sites of carotid plaque rupture, significant ^18^F-NaF uptake was observed, and was associated with histological evidence of active calcification, macrophage infiltration, apoptosis and necrosis [96]. Coronary atherosclerotic plaques with focal ^18^F-NaF uptake were seen in 18 patients (45%) with stable angina. Intravascular ultrasonography in these patients revealed more high-risk features in plaques with increased ^18^F-NaF uptake, such as positive remodelling, microcalcification and necrotic core, than in plaques without ^18^F-NaF uptake. These promising results indicate, that ^18^F-NaF PET imaging might be a sensitive method for identifying and localizing ruptured and high-risk coronary plaques.

**Fig. 3:**
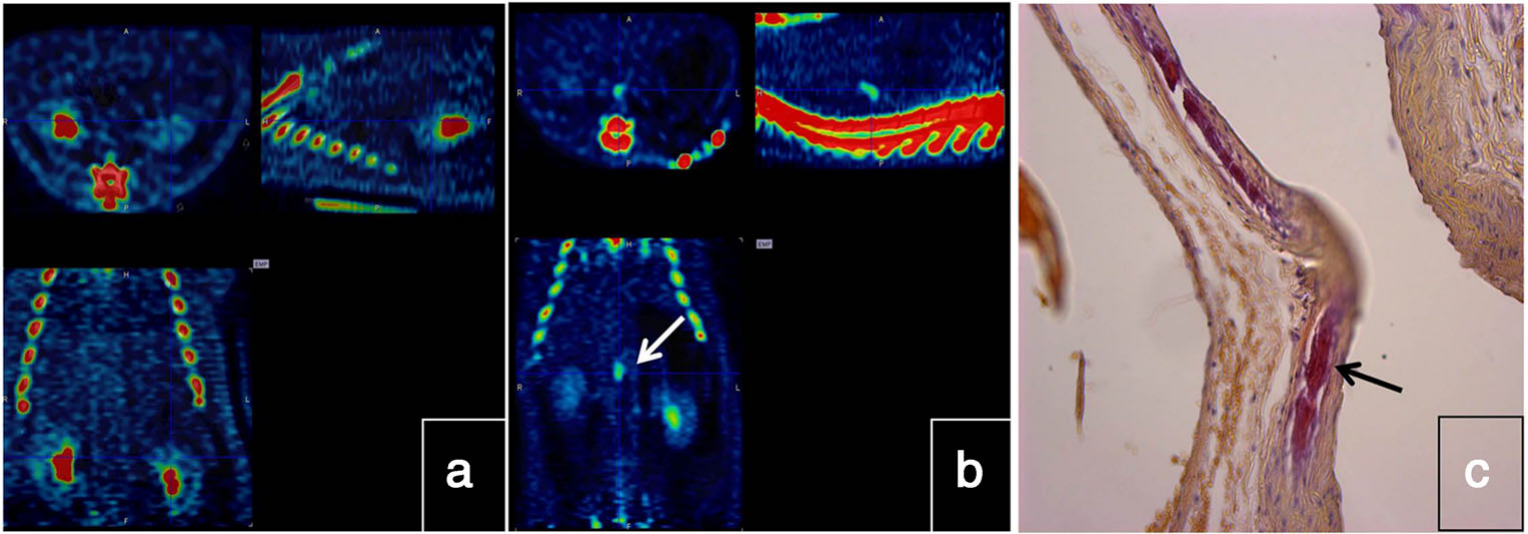
Sprague Dawley rats at 10–12 weeks of age were subjected to either a chow diet or a chow diet supplemented with warfarin (3 mg/g + 1.5 mg/g K1) for 2 weeks. **a** **b** ^18^F-NaF PET/CT images obtained after 2 weeks in animals on the chow diet (**a**) and in animals on the warfarin-supplemented chow diet (**b**): animals on the supplemented diet show uptake in the descending aorta indicating hotspots of calcification which are not seen in the animals on the chow diet. **c** Ex vivo histochemical Alizerin Red staining of aortic tissue reveals calcification of the vasculature

Irkle et al. reported intriguing results of an extensive analysis of vascular ^18^F-NaF binding using electron microscopy, autoradiography, histology and preclinical and clinical PET/CT [97]. ^18^F-NaF was found to directly adsorb to calcified areas in mineralized vascular tissue and this binding was found to be highly specific, as ^18^F-NaF uptake was seen solely in calcification with no localization in other soft tissues. Furthermore, ^18^F-NaF uptake was found to be highly dependent on the surface area of the calcification. The tracer was only absorbed to the outer layer of calcification regions, suggesting that ^18^F-NaF only detects active mineralization [97]. Fiz et al. found that ^18^F-NaF hot spots were present in atherosclerotic plaques in 86% of patients at sites without visible calcification on CT [98]. These findings suggest that ^18^F-NaF localizes at sites of microcalcification that are invisible on CT at the current clinical resolution, and strongly suggest that microcalcifications are a cause of atherosclerosis rather than a consequence [2]. Additionally, mice treated with warfarin (vitamin K antagonist) showed ^18^F-NaF hotspots in the abdominal aorta within 2 weeks of treatment, indicating that drug-induced inactivation of vitamin K-dependent proteins results in early active mineralization (unpublished data; Fig. 3).

The characteristic properties of ^18^F-NaF as a highly specific ligand for the detection of pathologically high-risk microcalcification, and therefore early unstable atherosclerotic disease, makes it a highly valuable tracer for noninvasive evaluation in human high-risk atherosclerotic plaques.

## The future: novel PET radiotracers

### ^18^F-Aluminium labelling

For routine PET imaging, ^18^F is today the best radionuclide with a half-life (*T*_½_) of 109.8 min and low β^+^ energy (0.64 MeV). Compared with other short-lived radionuclides, such as ^11^C (*T*_½_ = 20.4 min), its half-life is long enough to allow synthesis and imaging procedures to be extended over hours, enabling kinetic studies and high-quality metabolite and plasma analysis.

Efficient ^18^F labelling of peptides and proteins is often a multistep process involving labelling and purification of a prosthetic group (synthon) and subsequent conjugation of the ^18^F-labelled synthon to the peptide/protein with or without activation. If necessary, the ^18^F conjugate is purified by a final purification step. Over the years, a variety of prosthetic groups have been developed ranging from amine-reactive groups such as *N*-succinimidyl-4-[^18^F]fluorobenzoate ([^18^F]SFB) [99] to chemoselective groups, for example 4-[^18^F]fluorobenzaldehyde and ^18^F-FDG [100, 101], that react with an aminooxy-modified or hydrazine-modified peptide.

A special prosthetic group approach that has been explored in ^18^F labelling chemistry is click chemistry methodology. Click chemistry appears to be an effective method for radiolabelling peptides and proteins, because the click reaction is fast, bioorthogonal, chemoselective and regioselective, and results in relatively high yields and can be performed in aqueous media. This copper(I)-catalysed azide-alkyne cycloaddition reaction has been exploited in radiopharmaceutical chemistry by several research groups [101–106]. A variant of the copper(I)-catalysed click reaction is the copper-free click reaction that does not require the use of the cytotoxic metal. Reactions of electron-deficient tetrazines with ring-trained trans-cyclooctenes or norbenes have been investigated [107–111]. Click reactions, Cu(I)-catalysed and copper-free, have been shown to be powerful and versatile reactions for the synthesis of ^18^F-labelled peptides and proteins.

Although there are a variety of possible methods for introducing ^18^F into a peptide or protein, a major drawback of the ^18^F-labelling methods described above is that they are labourious (require azeotropic drying of the fluoride and multiple purification steps) and thus time-consuming. In search of a kit-based ^18^F-labelling method, new ^18^F-labelling strategies based on fluorine–silicon [112–115], fluorine–boron [116–118], and fluorine–phosphorus [119] have been developed. A straightforward chelator-based approach to labelling peptides and proteins with ^18^F was proposed in 2009 by McBride et al. [120]. They demonstrated that it is feasible to radiofluorinate compounds by first creating a stable ^18^F-Al complex which can subsequently be attached to the compound via a chelator. The ^18^F-Al labelling procedure has recently been significantly improved by optimizing chelators and labelling conditions [121]. This ^18^F-labelling technique has been demonstrated to be applicable to many peptides and proteins and has proved to be a versatile approach, especially for peptides and proteins that are not stable under harsh labelling conditions.

### Peptide-based and protein-based PET radiotracers

^18^F-NaF has excellent pharmacokinetic properties (i.e. fast blood clearance, urinary excretion, high uptake in regenerating bone, minimal binding to serum proteins) and in 1972 was approved by the US Food and Drug Administration for use as a clinical PET tracer [122]. Although the studies discussed above show that ^18^F-NaF is a suitable tracer for the detection of (early) vascular microcalcifications, uptake of ^18^F-NaF in these lesions is not specific, hampering elucidation of pathological molecular mechanisms. To define potential treatment targets based on underlying pathophysiological mechanisms, radiotracers that specifically bind to vascular calcification-specific biomarkers (i.e. receptors, enzymes, proteins) are necessary. In addition, it is difficult to distinguish intimal from medial calcification with noninvasive imaging techniques and specific radiotracers are necessary to differentiate between these two vascular calcification entities.

To investigate and decipher the underlying molecular mechanisms that result in vascular calcification, design and synthesis of smart diagnostic nuclear imaging probes is an appropriate approach. Multimodality molecular imaging now plays an important role in preclinical research as it combines the strengths of different imaging modalities to elucidate molecular basics of diseases, e.g. PET, single photon emission tomography (SPECT), fluorescence, and contrast-enhanced MRI. Specific imaging probes which combine optical imaging with nuclear medicine imaging modalities (SPECT/CT and PET/CT) are under development and enable additional ex vivo tissue analysis using microscopy to analyse lesions at the (sub)cellular level after a SPECT/CT or PET/CT scan. These multimodal tracers will mainly be used in preclinical imaging studies. There is also a role for these probes in clinical studies during intraoperative surgery.

During recent decades, the utility of radiolabelled peptides and proteins in preclinical and clinical research studies has been proven. Solid-phase peptide synthesis (SPPS) is currently the preferred technique for the production of synthetic peptides and proteins. Chemical protein synthesis allows unlimited variation of the polypeptide chain by incorporation of nonnatural amino acids such as d-amino acids, and fluorescent or affinity tags, chelators for metal ions or radioisotopes for use in MRI, PET and SPECT, at single specific sites. Selective protein modification at single sites cannot be achieved through regular labelling of biologically obtained proteins. Current optimized SPPS chemistry protocols enable effective peptide synthesis of 30–50 amino acids. For the synthesis of peptides and proteins larger than 30–50 amino acids, breakthrough ‘native chemical ligation’ (NCL) technologies have been developed that enable the formation of a peptide bond between two unprotected peptides resulting in larger synthetic proteins (50–200 amino acids) with a fully native peptide backbone [123, 124]. NCL can also be used to efficiently introduce labels into chemically synthesized proteins [125].

**Fig. 4:**
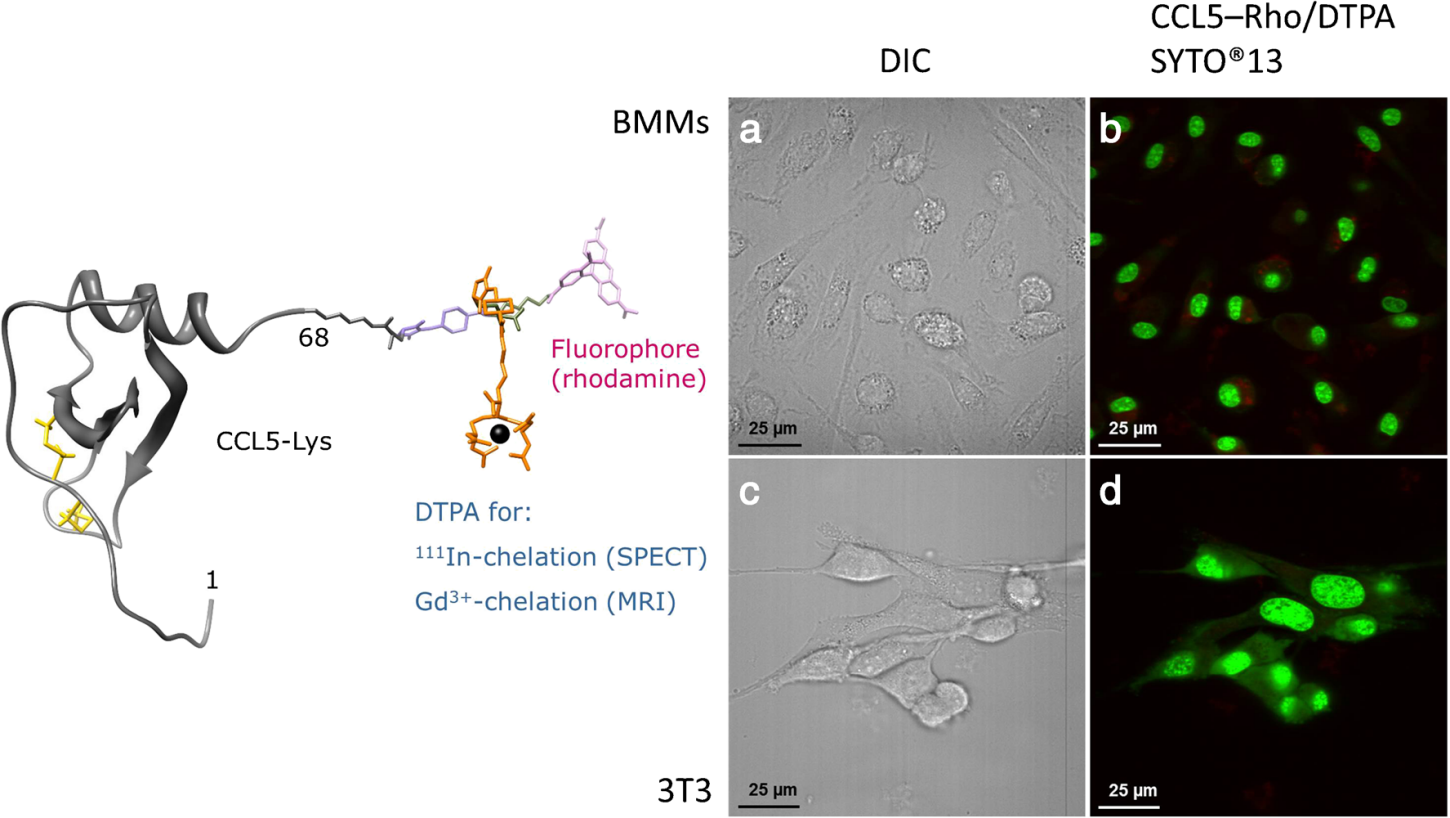
*Left*: Model of chemokine CCL5 synthesized by Boc-based SPPS and NCL. An extra lysine residue was coupled at the C-terminus of CCL5. Subsequently, a bimodal rhodamine/DTPA label was conjugated through an oxime bond. *Right*: Confocal microscopy imaging of mouse bone marrow derived macrophages (*BMMs*) and 3T3 fibroblasts. **a** DIC contrast image of BMMs; **b** Merged image of BMMs stained with CCL5–rhodamine/DTPA (1,500 nM) and with SYTO13 (2 μM; nuclei staining); **c** DIC contrast image of 3T3 fibroblasts; **d** Merged image of 3T3 fibroblasts stained with CCL5–rhodamine/DTPA (1,500 nM) and SYTO13 (2 μM)

For site-specific conjugation of a multimodal label (fluorophore and chelator; Fig. 4) to a protein, two strategies have been designed and optimized by our research group. One strategy uses a thiaproline residue appended to a lysine sidechain (Lys[Thz]), as an unlockable thiol handle that enables orthogonal modification of prefolded proteins [126]. This method is compatible with Boc-based SPPS, NCL, and standard methods for disulfide bridge formation. The other strategy is based on aniline-catalysed oxime bond formation [127]. Oxime ligations comprise chemoselective mild reactions of a ketone or aldehyde with an aminooxy to form oximes which are stable at neutral pH [128]. Because the widely used levulinoyl ketone group undergoes intramolecular rearrangement, a novel oxime ligation strategy has been developed with 3-(2-oxopropyl)-benzoic acid as a ketone moiety that appears to give higher oxime ligation rates and yields [129].

**Fig. 5:**
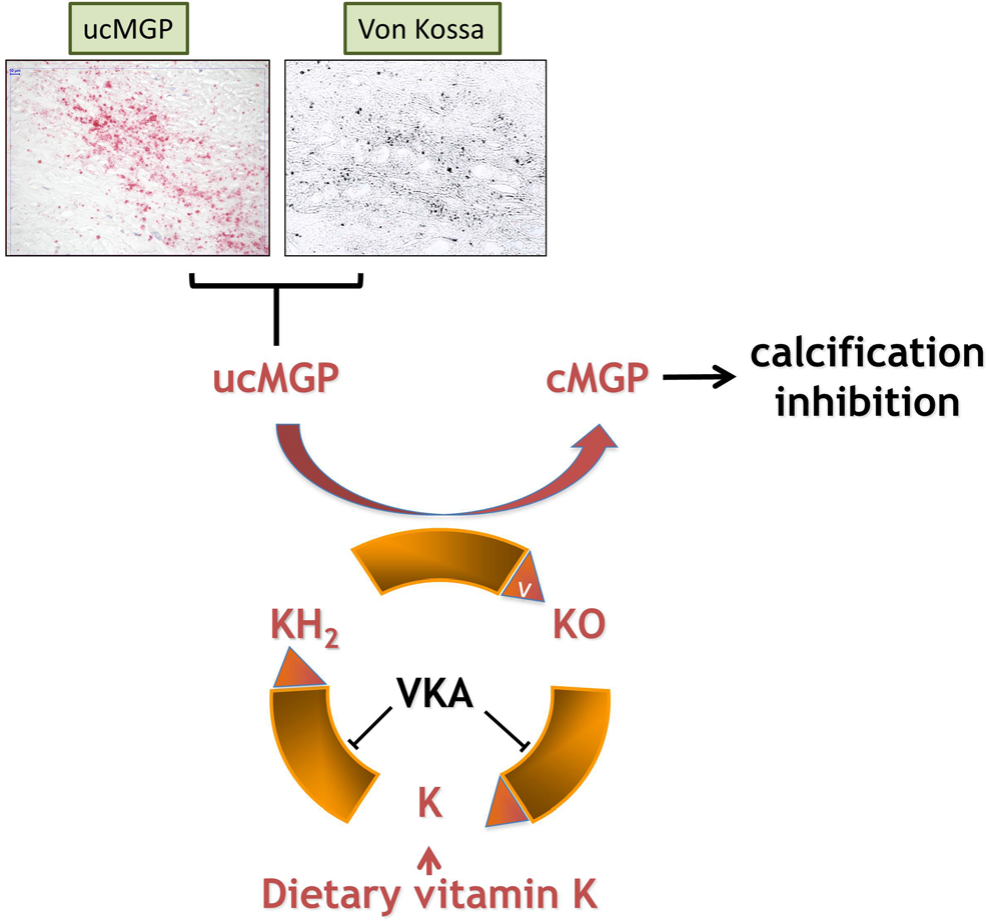
Vitamin K metabolism in the secretion of vitamin K-dependent MGP. Scheme of the vitamin K cycle: in the endoplasmic reticulum, posttranslational modification of Glu to Gla residues via reduction of vitamin K to the hydroquinone form (KH_2_). KH_2_ is oxidized to KO by the enzyme GGCX thereby facilitating the carboxylation of ucMGP to cMGP. The enzyme VKOR recycles KO back to K and KH_2_ so that vitamin K can be used some 1,000 times. VKA inhibits VKOR and thus the recycling of vitamin K. This causes a vitamin K deficiency with subsequent ucMGP formation. UcMGP is inactive thereby allowing the formation of microcalcifications

The techniques described above are used and will be used to synthesize and radiolabel (multimodal) peptide-based molecular imaging agents. An interesting biomarker of vascular calcification is matrix Gla protein (MGP; Fig. 5) [16]. This protein consists of 84 amino acids and requires vitamin K-dependent gamma-carboxylation for its function [130]. It is mainly expressed by chondrocytes, VSMCs, endothelial cells and fibroblasts. MGP binds calcium crystals, inhibits crystal growth and plays a role in preventing osteoblastic differentiation of normal VSMCs [131, 132]. As MGP binds calcium crystals, it may be a suitable molecular imaging probe for the investigation of early vascular calcium deposits. Moreover, since uncarboxylated MGP seems to precede microcalcification, detection of inactive MGP might be an interesting alternative [2, 133].

Another interesting biomarker in vascular calcification is bone morphogenetic protein-2 (BMP-2). BMP-2 is a 114 amino acid protein which transforms undifferentiated cells and subpopulations of VSMCs into bone-forming cells [134, 135]. BMP also binds calcium crystals and thus can be used as a molecular imaging probe. Osteocalcin (OC) is another vitamin K-dependent protein that can be used as a molecular imaging probe for vulnerable plaque [136]. This 49 amino acid protein inhibits calcium salt precipitation in vitro [137] and is strongly upregulated in advanced atherosclerotic lesions [138].

As well as the proteins discussed above, there are other promising imaging probe candidates. Antibodies or preferably antibody fragments that specifically bind to these proteins are of great interest for the development of suitable tracers for molecular imaging of molecular processes early in the initiation of microcalcification. Moreover, peptides and proteins that bind to receptors, enzymes or proteins that are upregulated during vascular calcification are promising candidates. The development of new imaging agents requires a multistep approach, including target selection, organic synthesis of molecular imaging agents, optimization of target affinity and pharmacokinetic profile of the tracer, and preclinical evaluation.

## Conclusion

**Fig. 6:**
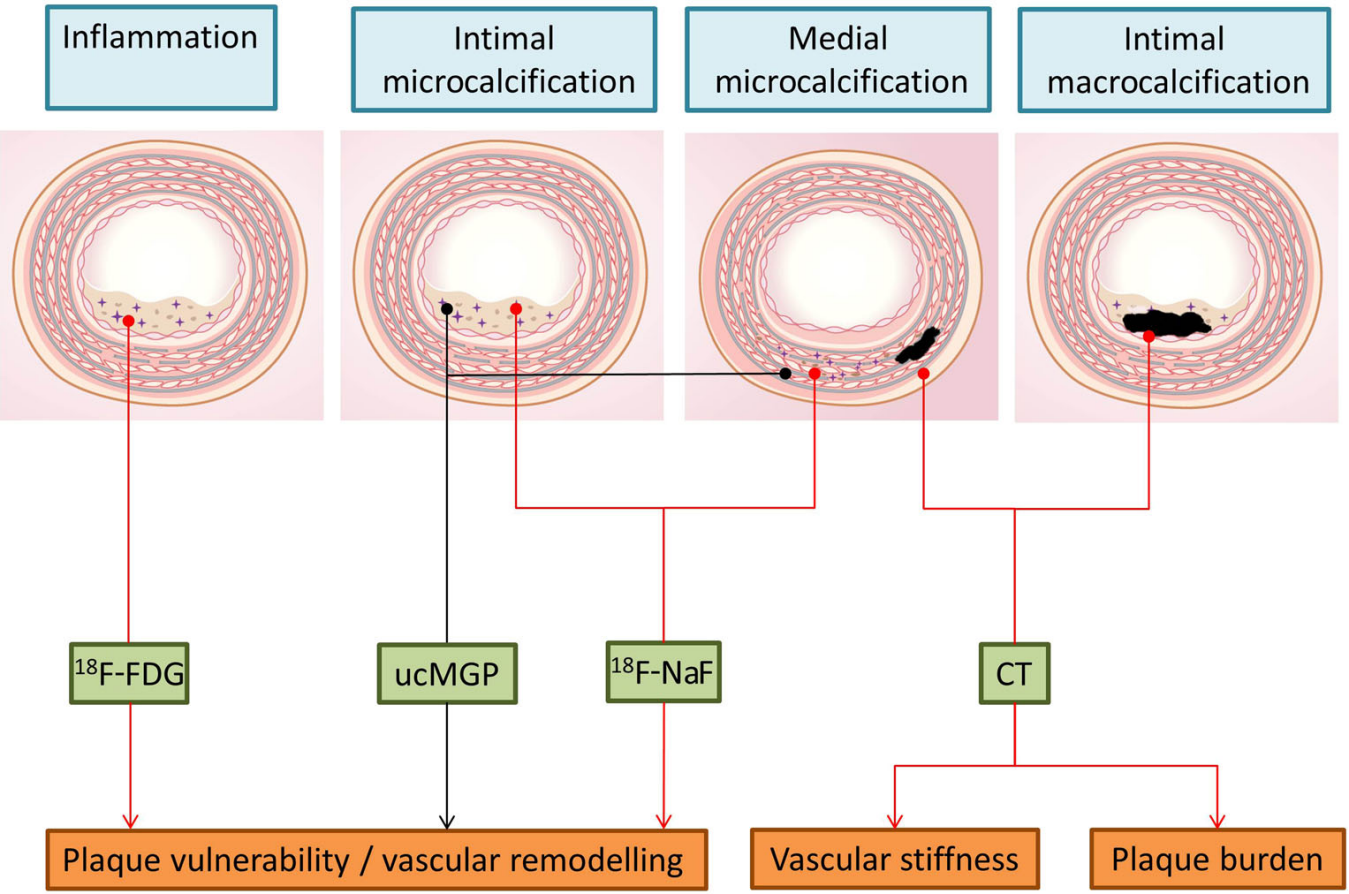
Different types of vascular calcification can occur in the vasculature. Vascular calcification is clinically measured by CT and represents the amount of vascular burden. Moreover, calcification detected on CT in the media relates to vascular stiffness. However, microcalcification cannot be visualized by CT and these early stages of calcification can now be detected by ^18^F-NaF PET. Microcalcifications are responsible for destabilizing the plaque, causing an increased risk of plaque rupture. They also cause vascular remodelling when present in the vascular media. The measurement of inactive MGP as a marker of increased risk of the formation of microcalcifications is currently under development

Atherosclerosis is a multifactorial process that involves both local and systemic processes. The vulnerable atherosclerotic plaque cannot simply be detected by plaque size, and thus identification of molecular and cellular processes underlying plaque vulnerability is crucial for diagnosis and treatment (Fig. 6). Recent progress in imaging has improved detection of atherosclerotic lesions and has led to the ability to screen for vulnerable atherosclerotic plaques. Combining expertise in biology, chemistry, nuclear medicine and cardiology will advance our understanding of critical determinants of the high-risk plaque and result in tailor-made imaging radiotracers to differentiate the vulnerable plaque. Ultimately, this strategy will open novel avenues for diagnosis and treatment of the patient with high-risk disease.
